# Fully automated landmarking and facial segmentation on 3D photographs

**DOI:** 10.1038/s41598-024-56956-9

**Published:** 2024-03-18

**Authors:** Bo Berends, Freek Bielevelt, Ruud Schreurs, Shankeeth Vinayahalingam, Thomas Maal, Guido de Jong

**Affiliations:** 1 3D Lab Radboudumc, Radboud University Medical Center, Geert Grooteplein Zuid 10, 6525 GA Nijmegen, The Netherlands; 2 Department of Oral and Maxillofacial Surgery, Amsterdam University Medical Center (UMC), AMC, Academic Center for Dentistry Amsterdam (ACTA), Meibergdreef 9, 1105 AZ Amsterdam, The Netherlands; 3 Department of Oral and Maxillofacial Surgery, Radboud University Medical Center Nijmegen, Geert Grooteplein Zuid 10, 6525 GA Nijmegen, The Netherlands

**Keywords:** Deep learning, DiffusionNet, Cephalometry, Landmarks, 3D photogrammetry, 3D meshes, Computational science, Genetics research, Dentistry, Three-dimensional imaging

## Abstract

Three-dimensional facial stereophotogrammetry provides a detailed representation of craniofacial soft tissue without the use of ionizing radiation. While manual annotation of landmarks serves as the current gold standard for cephalometric analysis, it is a time-consuming process and is prone to human error. The aim in this study was to develop and evaluate an automated cephalometric annotation method using a deep learning-based approach. Ten landmarks were manually annotated on 2897 3D facial photographs. The automated landmarking workflow involved two successive DiffusionNet models. The dataset was randomly divided into a training and test dataset. The precision of the workflow was evaluated by calculating the Euclidean distances between the automated and manual landmarks and compared to the intra-observer and inter-observer variability of manual annotation and a semi-automated landmarking method. The workflow was successful in 98.6% of all test cases. The deep learning-based landmarking method achieved precise and consistent landmark annotation. The mean precision of 1.69 ± 1.15 mm was comparable to the inter-observer variability (1.31 ± 0.91 mm) of manual annotation. Automated landmark annotation on 3D photographs was achieved with the DiffusionNet-based approach. The proposed method allows quantitative analysis of large datasets and may be used in diagnosis, follow-up, and virtual surgical planning.

## Introduction

The fields of genetics, orthodontics, craniomaxillofacial surgery, and plastic surgery have greatly benefitted from advances in imaging technology, particularly in three-dimensional (3D) imaging. Within these fields, cone-beam computed tomography (CBCT) has become a well-established imaging technique as an alternative for computed tomography (CT) imaging, the conventional imaging method for depicting hard tissues^1^. CBCT provides a high-resolution multiplanar reconstruction of the craniofacial skeleton and facial soft tissue. However, a drawback of CT and CBCT is the use of ionizing radiation. In contrast, 3D stereophotogrammetry can capture a detailed and accurate representation of craniofacial soft tissue without the use of ionizing radiation and, therefore, has gained popularity in soft-tissue analysis^2–4^.

Cephalometric analysis can be performed on 3D stereophotographs to extract information about the position of individual landmarks or distances and angles between several landmarks, with the purpose of objectifying clinical observations^5^. Despite being a commonly used diagnostic tool in the craniofacial region, landmarking often remains a manual task that is time-consuming, prone to observer variability, and affected by observer fatigue and skill level^6,7^. Therefore, there has been a growing interest in using artificial intelligence (AI), such as deep learning and machine learning algorithms to automate the landmark identification process.

Several studies have described the use of deep learning algorithms for the automation of hard-tissue landmark extraction for cephalometric analysis^5,8^. Studies that include soft-tissue landmarks utilize (projective) 2D imaging, are pose dependent, or require manual input^9,10^. Only a limited number of studies were performed on the automated extraction of facial soft-tissue landmarks from 3D photographs^11,12^. Nevertheless, since the studies in question did not integrate deep learning algorithms into their methodologies, and considering that deep learning has demonstrated enhanced accuracy in hard tissue landmarking, the effectiveness of soft tissue landmarking could potentially see improvements through the integration of deep learning techniques^5^. Therefore, this study aimed to develop and validate an automated approach for the extraction of soft-tissue facial landmarks from 3D photographs using deep learning.

## Material and methods

### Data acquisition

In total, 3188 3D facial photographs were collected from two databases: the Headspace database (n = 1519) and the Radboudumc’s database (n = 1669)^13,14^. The Radboudumc’s data consisted of healthy volunteers (n = 1153) and Oral and Maxillofacial Surgery patients (n = 516). The Radboudumc dataset was collected in accordance with the World Medical Association Declaration of Helsinki on medical research ethics. The following ethical approvals and waivers were used: Commissie Mensgebonden Onderzoek (CMO) Arnhem-Nijmegen (Nijmegen, the Netherlands) #2007/163; ARB NL 17934.091.07; CMO Arnhem-Nijmegen (Nijmegen, the Netherlands) #2019-5793. All data were captured using 3dMD’s 5-pod 3dMDhead systems (3dMDCranial, 3dMD, Atlanta, Georgia USA). Exclusion criteria were large gaps within the mesh, stitching errors, excessive facial hair interfering with the facial landmarks, meshes that lacked texture (color information), and mesh-texture mismatches. An overview of the data is presented in Table 1.

**Table 1:** Dataset distribution with the percentage of the total dataset.

Dataset	Train		Test		Total	
	n	%	n	%	n	%
Headspace	1053	36.3	192	6.6	1245	43.0
Radboudumc control	974	33.6	165	5.7	1139	39.3
Radboudumc patient	436	15.1	77	2.7	513	17.7
Total	2463	85	434	15	2897	100

### Data annotation

The 3D photographs were manually annotated by a single observer using the 3DMedX^®^ software (v1.2.29.0, 3D Lab Radboudumc, Nijmegen, The Netherlands; details can be found at https://3dmedx.nl). The following ten cephalometric facial landmarks were annotated: exocanthions, endocanthions, nasion, nose tip, alares, and cheilions. The 3D landmarks were annotated on the mesh surfaces (faces) independent of vertex coordinates. The texture of the 3D photographs was used in the annotation process as a visual cue. Manual annotation was repeated on 50 randomly selected 3D photos by the first observer, a second observer, and a third observer to assess the intra-observer and inter-observer variability.

### Automated landmarking workflow

**Figure 1:**
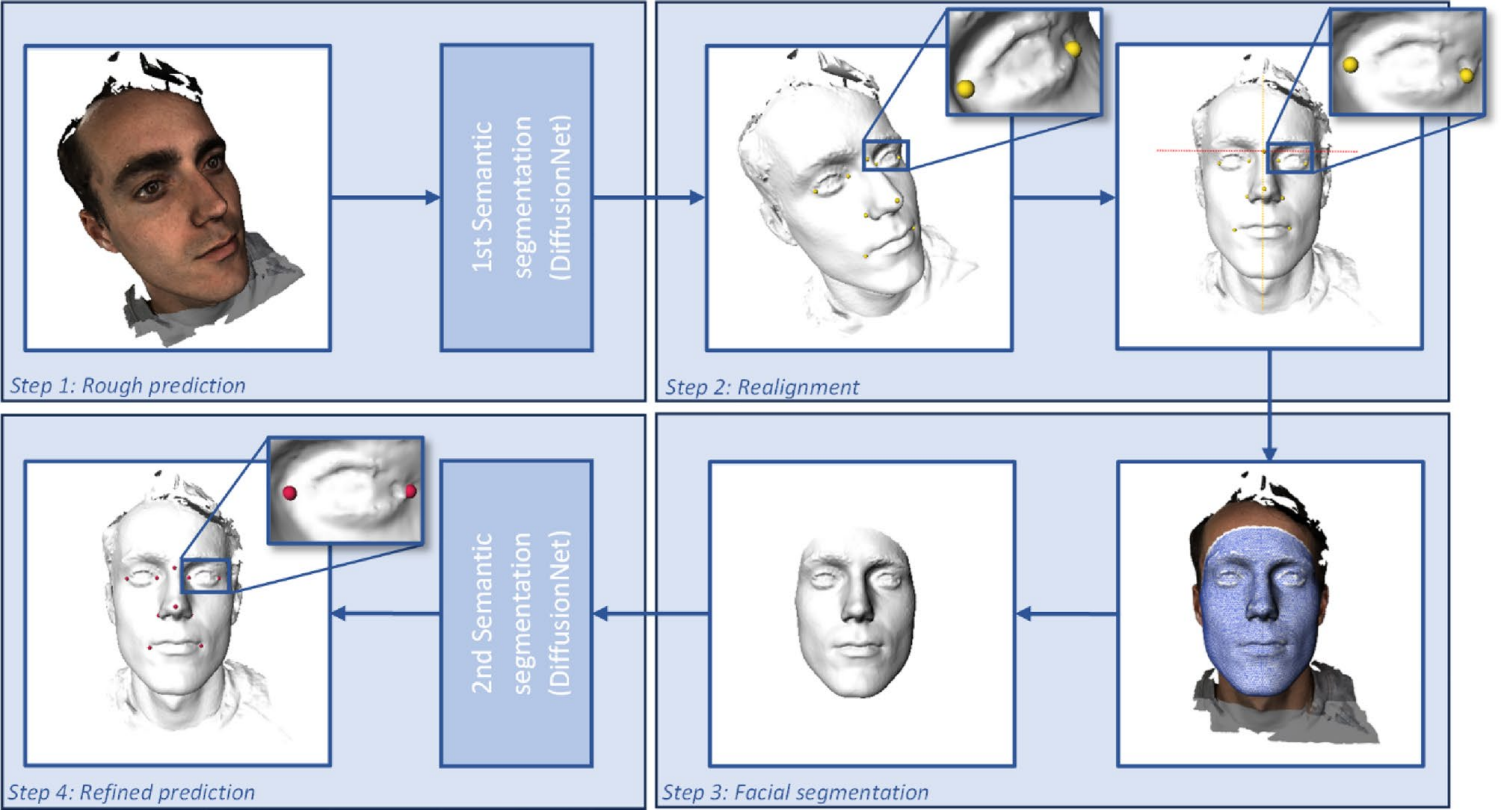
Automated landmarking workflow. Step 1: First semantic segmentation task for rough landmark prediction. Step 2: Realignment of the meshes using the roughly predicted landmarks. Step 3: Facial region segmentation (white) using MeshMonk (blue wireframe). Step 4: Second semantic segmentation task for refined landmark prediction.

The automated landmarking workflow was developed for the same cephalometric landmarks (exocanthions, endocanthions, nasion, nose tip, alares, and cheilions). It consisted of four main steps: (1) rough prediction of landmarks using an initial DiffusionNet on the original meshes; (2) realignment of the meshes based on the roughly predicted landmarks; (3) segmentation of the facial region through fitting of a template facial mesh using a morphable model; (4) refined landmark prediction on the segmented meshes using a final DiffusionNet^15^. The DiffusionNet models used spatial features only and did not use texture information for the automated landmarking task. An overview of the workflow can be seen in Fig. 1.

### DiffusionNet

DiffusionNet is a deep learning algorithm able to learn upon non-uniform geometric data, like 3D curved surfaces. It is designed to address challenges associated with the representation and analysis of surfaces in the field of geometric deep learning. DiffusionNet was used for this study as it is a highly accurate method for performing segmentation tasks, also when compared to other state-of-the-art mesh-based deep-learning algorithms. A key feature is the relative mesh sampling/resolution robustness of the algorithm as well as the invariance to input orientations. The DiffusionNet consists of three main components: a multilayer perceptron (MLP) for feature transformation, a diffusion layer responsible for information propagation, and a spatial gradient enabling the network to understand and learn. The architecture of the DiffusionNet depends on the configuration of several adjustable parameters. These include C-width, which corresponds to the size of the DiffusionNet and N-blocks, corresponding to the number of DiffusionNet blocks. In addition, the size of the (per-vertex) MLP can be altered by adjustment of its layer size^15^.

### Training

The data were randomly divided into two sets, 85% for training and 15% for testing of the DiffusionNet models. As a data augmentation step, the 3D meshes from the training dataset were mirrored over the YZ plane to double the number of scans available for training. No validation set was used during training.

### Step 1: Rough prediction of landmarks

**Figure 2:**
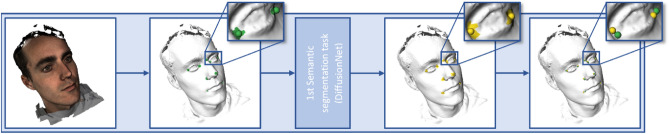
First semantic segmentation task. The manually annotated landmarks (spheres) and corresponding masks are visualized in green. The green areas represent the vertices within 5 mm of the manually annotated landmark. The roughly predicted landmarks are visualized in yellow. The yellow area represents the positively predicted vertices out of which the rough landmarks will be calculated.

A DiffusionNet was utilized for initial prediction of the exocanthions, endocanthions, nasion, nose tip, alares, and cheilions as visualized in Fig. 2^15^.

#### Preprocessing

To speed up the training process for rough landmark prediction, each mesh was downsampled to a maximum of 25.000 vertices^16^. After downsampling, the 3D meshes had a mean inter-vertex distance of 2.035 ± 0.356 mm. Subsequently, a mask was applied, assigning a value of 1 to all vertices located within 5 mm Euclidean distance to the manually annotated landmarks and a value of 0 to the remaining vertices.

#### DiffusionNet configuration for the first semantic segmentation task

Six output channels were configured for the first semantic segmentation task. The two midsagittal landmarks (nasion and nose tip) were assigned an individual channel. The four bilateral landmark pairs were assigned to the four remaining channels. The DiffusionNet model was configured with a C-width of 256, an MLP of 256 and 256 channels, and an N-block of 12. The network used an Adam optimizer with a Cosine Annealing learning rate of 2 × 10^−5^ and a T_max_ of 50 epochs, representing the number of epochs at which the learning rate is annealed to its minimum and starts increasing again. Furthermore, a binary cross-entropy loss and a dropout rate of 0.10 were applied. Since the orientation and position of the included 3D meshes was not fixed, the network was trained with Heat Kernel Signature (HKS) Features of the 3D meshes. The final output layer was linear. The model was implemented in PyTorch on a 24 GB NVIDIA RTX A5000 GPU and trained for 200 epochs.

#### Post-processing

After the semantic segmentation, the model was used to predict which vertices belonged to each of the configured channels. For the symmetrical landmarks, a 3D clustering algorithm was utilized to distinguish the predicted vertex clusters from each other. Subsequently, a weighted combination of the output values (activations), as well as the locations of each of the vertices that received a non-zero activation value, were used to determine the landmark positions using Eq. 1.

1 $$Predicted\;landmark\;location = \frac{{\sum \left( {10^{{Activations_{1,2, \ldots ,i} }} *coordinate_{1,2, \ldots ,i} } \right)}}{{\sum 10^{{Activations_{1,2, \ldots ,i} }} }}$$

A plane was formed by connecting the predicted nasion, nose tip, and cheilion midpoint to establish if the bilateral landmarks were on the left or right side of the face using the plane equation.

### Step 2: Realignment

Based on the rough prediction of the exocanthions, nasion, and cheilions, the 3D meshes were positioned in a reference frame. The nasion was defined as the origin, with the x-axis running parallel to the line connecting both exocanthions and the z-axis parallel to the nasion-cheilion midpoint line (Fig. 1).

### Step 3: Facial region segmentation

Facial segmentation was used to standardize the realigned 3D meshes for refined landmark prediction and reduce processing time without the need for downsampling. The MeshMonk algorithm, which utilizes a combination of rigid and non-rigid template matching, was utilized for segmentation (implemented in C++)^10^. The default face template of MeshMonk was used. The exocanthions, nose tip, and cheilions (step 2) were used for the initial registration of the facial template mesh to the 3D meshes. The configuration of the MeshMonk fitting algorithm is given in the Appendix. An additional margin of 15 mm around the fitted MeshMonk template was included to supply the final DiffusionNet with more context while still excluding hair regions (Fig. 1). After facial segmentation, the mean inter-vertex distance of the 3D meshes was 1.39 ± 0.16 mm.

Besides facial segmentation, the MeshMonk algorithm can also be utilized for landmark annotation. The ten landmarks were collected using this semi-automatic approach to serve as a reference for the precision of the automated annotation approach. In contrast to the automated approach, the manually annotated landmarks were used for template fitting in the semi-automated approach to comply with the MeshMonk workflow^10^.

### Step 4: Refined landmark prediction

**Figure 3:**
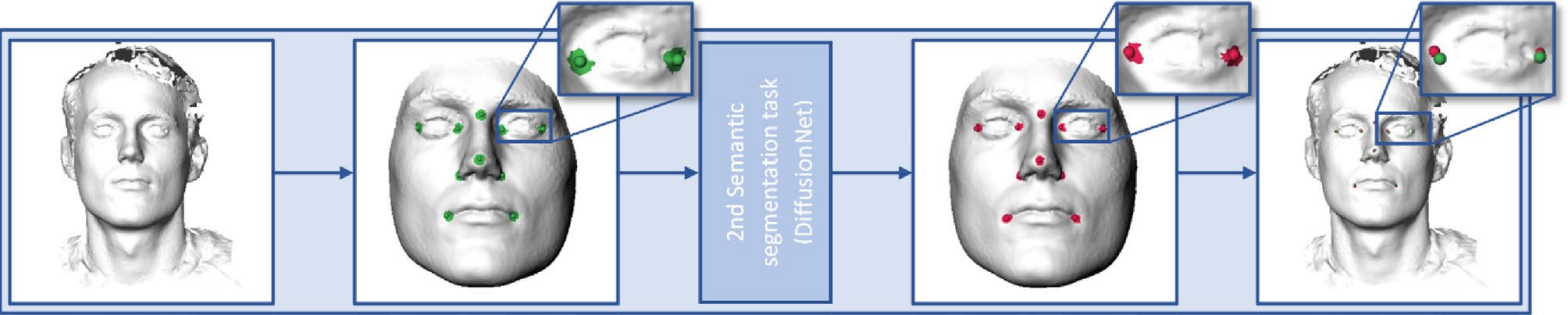
Second semantic segmentation task. The manually annotated landmarks and corresponding masks that were used for training are visualized in green. The green areas represent the vertices within 3.5 mm of the manually annotated landmark. The positively predicted vertices and the calculated refined predicted landmarks are visualized in red.

A second semantic segmentation task, using DiffusionNet, was used to predict the landmarks on the realigned and segmented 3D meshes (Fig. 3).

#### Preprocessing

In contrast to step 1, the meshes were not downsampled as it is made unnecessary by the facial region segmentation step. A mask was created in which the vertices within 3.5 mm of the manually annotated landmarks were assigned value 1 and the other vertices were assigned value 0. Default mesh normalization and scaling were applied as provided by the DiffusionNet package^15^.

#### DiffusionNet configuration for the second semantic segmentation task

For the second semantic segmentation task, ten output channels were configured: each individual landmark was assigned to an individual channel. The DiffusionNet was configured with a C-width of 384, an MLP of 768, and an N-blocks of 12. Compared to the first network, the same optimizer, loss, and drop-out were used. However, this second network was trained with XYZ settings instead of HKS settings as the rotation invariance was no longer present after step 3 (Fig. 1). The model was implemented in PyTorch on a 24 GB NVIDIA RTX A5000 GPU and trained for 200 epochs. The final output layer was linear.

#### Post-processing

A weighted combination of the activations, supplemented by the locations of each of the vertices, was again used to determine the final landmark positions.

### Statistical analysis

Statistical analyses were performed on available patient characteristics to assess differences between the source databases and between the training and test data. To assess the intra-observer and inter-observer variability of the manual annotation method, the Euclidean distances between the landmarks annotated by the different observers were calculated. Descriptive statistics were used to summarize the results. The Euclidean distances between the predicted and the manually annotated landmarks were calculated for every test set to evaluate the performance of automated landmarking; descriptive statistics were used for summarizing the results. This was done for both the rough (initial DiffusionNet) and the refined (final DiffusionNet) predictions. The performance of the automated landmarking workflow was compared to the intra-observer and inter-observer variability of the manual annotation method.

The Euclidean distances between the manually annotated landmarks and the predictions by the semi-automated MeshMonk method were calculated and compared to the precision of the refined predictions using a one-way repeated measures ANOVA test. A *p* value < 0.05 was used as a cut-off value for statistical significance.

### Ethical approval and Informed consent

CMO Arnhem-Nijmegen (Nijmegen, the Netherlands) #2007/163; ARB NL 17934.091.07; CMO Arnhem-Nijmegen (Nijmegen, the Netherlands) #2019-5793. Images of a 3D photo of the first author, who gave his informed consent to publish images in this online open-access publication, were used in Figs. 1, 2 and 3.

## Results

Based on the stated exclusion criteria, 291 3D photographs were excluded, yielding a total of 2897 3D photographs that were used for training and testing of the developed workflow (Table 1). Most of the exclusions were due to the lack of texture information (n = 271). The age and gender characteristics are given in Table 2. A statistically significant difference was found for age and gender between the source databases (*p* < 0.001 and *p* < 0.001, respectively). However, there were no statistically significant differences between ages and genders of the training and test splits (*p* = 0.323 and *p* = 0.479, respectively). There were no unknown genders or ages in the test dataset. The training dataset held one transgender case and had five unknown ages.

**Table 2:** Population characteristics per dataset.

Dataset	Age (years)				Gender		
	Mean	Std	Min	Max	Male	Female	Transgender
Headspace	35.9	17.6	2	90	631 (50.7%)	613 (49.2%)	1 (0.1%)
Controls	42.1	19.4	0	90	492 (43.2%)	647 (56.8%)	
Patients	27.8	10.9	13	69	190 (37.0%)	323 (63.0%)	

The intra-observer and interobserver differences of the manual annotation method are summarized in Tables 3 and 4, respectively. The overall mean intra-observer variability for manual annotation of the ten landmarks was 0.94 ± 0.71 mm; the overall mean interobserver variability was 1.31 ± 0.91 mm.

**Table 3:** The intra-observer variability for all ten landmarks is stated in millimeters ± standard deviation.

Exocanthion		Endocanthion		Nasion	Nose tip	Alare		Cheilion	
*Right*	*Left*	*Right*	*Left*			*Right*	*Left*	*Right*	*Left*
0.85 ± 0.65	0.87 ± 0.55	0.79 ± 0.88	0.79 ± 0.72	1.32 ± 1.04	0.88 ± 0.43	1.04 ± 0.53	0.99 ± 0.58	0.99 ± 0.79	0.85 ± 0.64

The intra-observer variability was determined by computing the Euclidean distance between annotations performed in twofold by a single observer.

**Table 4:** The interobserver variability is computed by comparing the Euclidean distance between annotations made by three different observers.

	Exocanthion		Endocanthion		Nasion	Nose tip	Alare		Cheilion	
	*Right*	*Left*	*Right*	*Left*			*Right*	*Left*	*Right*	*Left*
Observer 1 versus 2	1.16 ± 0.65	1.14 ± 0.67	1.08 ± 0.69	0.87 ± 0.58	1.64 ± 0.91	1.16 ± 0.59	1.34 ± 0.93	1.27 ± 0.79	0.93 ± 0.55	0.97 ± 0.66
Observer 1 versus 3	1.02 ± 0.85	0.95 ± 0.63	1.03 ± 0.80	1.08 ± 0.73	1.80 ± 1.20	1.77 ± 0.86	1.68 ± 1.19	1.35 ± 0.80	1.65 ± 1.04	1.43 ± 1.05
Observer 2 versus 3	1.31 ± 0.88	1.03 ± 0.57	0.97 ± 0.73	1.05 ± 0.64	2.21 ± 1.30	2.20 ± 1.07	1.33 ± 0.98	1.35 ± 0.82	1.27 ± 0.83	1.25 ± 0.84
Average	1.16 ± 0.80	1.04 ± 0.63	1.03 ± 0.74	1.00 ± 0.66	1.88 ± 1.17	1.71 ± 0.96	1.45 ± 1.05	1.32 ± 0.80	1.28 ± 0.88	1.22 ± 0.88

The Euclidean distances are stated in millimeters ± standard deviation.

The initial DiffusionNet showed an average precision of 2.66 ± 2.37 mm, and the complete workflow achieved a precision of 1.69 ± 1.15 mm. The performance of both models is summarized in Table 5. The workflow could be completed for 98.6% of the test data; for six 3D photos (1.4%), one of the rough landmarks required for the consecutive steps could not be predicted by the first DiffusionNet. Upon visual inspection, the six excluded 3D photos contained large gaps and/or substantial amounts of information outside the region of interest, such as clothing or hair. Since the workflow could not be completed, these data sets were excluded from the results.

**Table 5:** The precision of the rough (first DiffusionNet) and refined (second DiffusionNet) is determined by computing the Euclidean distance between the DiffusionNet-predicted and manually annotated landmarks and is stated in millimeters ± standard deviation.

	Exocanthion		Endocanthion		Nasion	Nose tip	Alare		Cheilion	
	*Right*	*Left*	*Right*	*Left*			*Right*	*Left*	*Right*	*Left*
Rough predictions	2.94 ± 2.38	2.86 ± 1.81	2.76 ± 2.40	2.83 ± 2.56	1.69 ± 1.05	1.58 ± 0.89	2.41 ± 1.98	2.52 ± 1.92	3.48 ± 3.67	3.51 ± 2.89
Refined predictions	2.25 ± 1.23	2.03 ± 1.27	1.37 ± 0.86	1.48 ± 1.00	1.48 ± 1.02	1.14 ± 0.73	1.79 ± 1.07	1.75 ± 1.11	1.71 ± 1.26	1.88 ± 1.34

**Figure 4:**
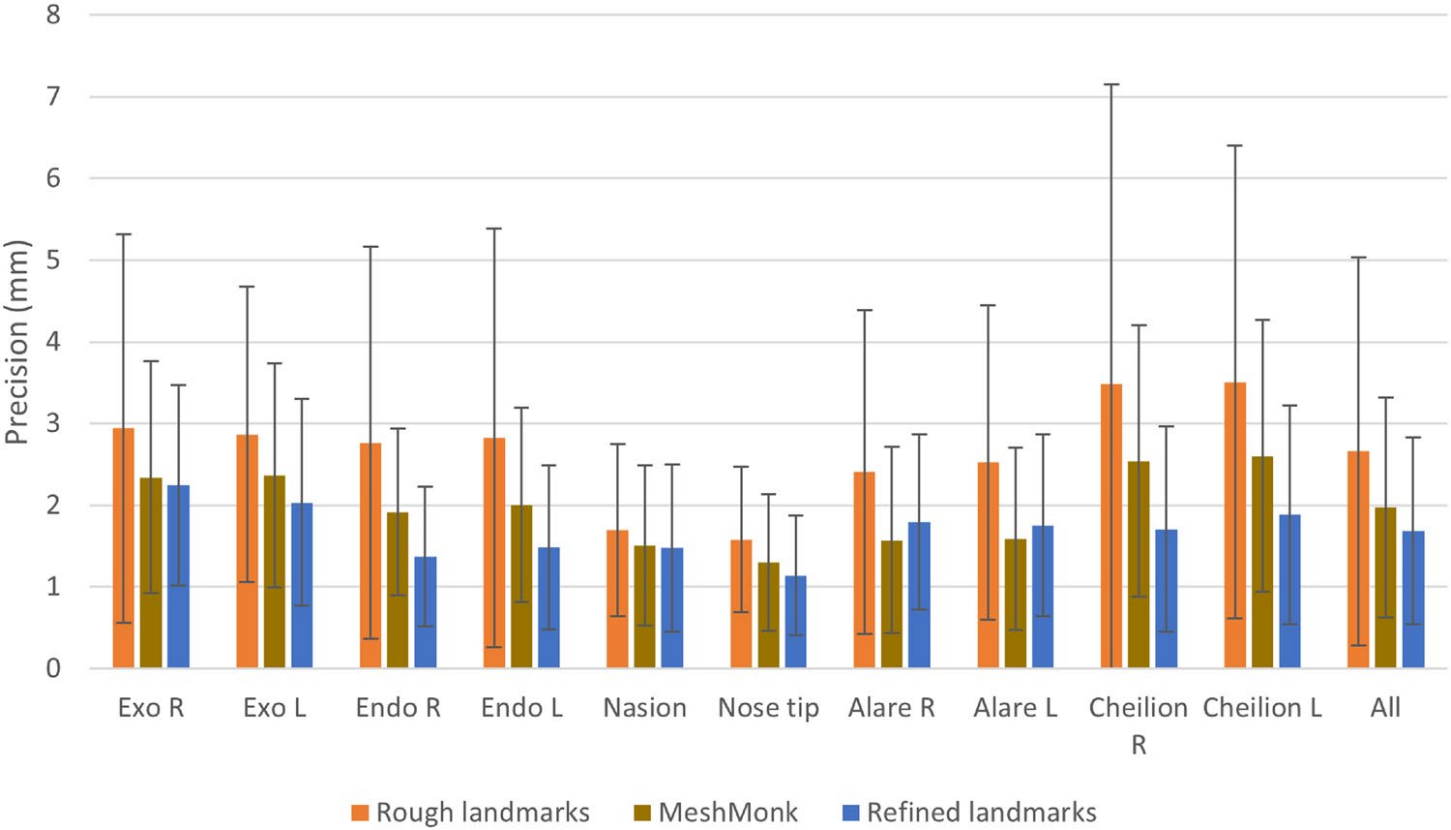
Precision of the landmarking methods. The precision of the prediction of the rough landmarks (first DiffusionNet), the refined landmarks (second DiffusionNet), and the semi-automated MeshMonk method are visualized for the right exocanthion (Exo R), left exocanthion (Exo L), right endocanthion (Endo R), left endocanthion (Endo L), nasion, nose tip, right alare (Alare R), left alare (Alare L), right cheilion (Cheilion R), and left cheilion (Cheilion L).

The precision was within 2 mm for 69% of the refined predicted landmarks, within 3 mm for 89% of the landmarks, and within 4 mm for 96% of the landmarks. Table 6 details the precision within these boundaries for the individual landmarks. The exocanthions and alares were found to perform the worst. The precision of the semi-automated MeshMonk method was on average 1.97 ± 1.34 mm for the ten landmarks (Fig. 4). Compared to this semi-automatic method, the DiffusionNet-based method was found to have significantly better precision for the left exocanthion, endocanthions, nose tip, and cheilions and worse precision for the alares; no significant differences were found for nasion and right exocanthion.

**Table 6:** Overview of the accuracy distribution of each landmark as predicted by the complete workflow.

	Percentage of landmarks predicted with a precision within range			
	< 2 mm (%)	< 3 mm (%)	< 4 mm (%)	< 5 mm (%)
Exocanthion right	47	77	90	97
Exocanthion left	56	80	82	97
Endocanthion right	80	96	99	100
Endocanthion left	76	92	98	99
Nasion	77	94	97	99
Nose tip	88	98	99	100
Alare right	62	88	96	99
Alare left	67	86	96	98
Cheilion right	71	89	95	97
Cheilion left	65	87	94	97
All Landmarks	69	89	96	98

## Discussion

Soft-tissue cephalometric analysis can be used to objectify the clinical observations on 3D photographs, but manual annotation, the current gold standard, is time-consuming and tedious. Therefore, this study developed a deep learning-based approach for automated landmark extraction from randomly oriented 3D photographs. The performance was assessed for ten cephalometric landmarks: the results showed that the deep-learning-based landmarking method was precise and consistent, with a precision that approximated the inter-observer variability of the manual annotation method. A precision < 2 mm, which may be considered a cut-off value for clinical relevance, was seen for 69% of the predicted landmarks^17,18^.

In the field of craniofacial surgery, different studies have applied deep-learning models for automated cephalometric landmarking, mainly focusing on 2D and 3D radiographs. Dot et al. used a SpatialConfiguration-Net for the automated annotation of 33 different 3D hard-tissue landmarks from CT images and achieved a precision of 1.0 ± 1.3 mm^19^. An automated landmarking method, based on multi-stage deep reinforcement learning and volume-rendered imaging, was proposed by Kang et al. and yielded a precision of 1.96 ± 0.78 mm^20^. A systematic review by Serafin et al. found a mean precision of 2.44 mm for the prediction of 3D hard-tissue landmarks from CT and CBCT images^5^.

Some studies did describe automated algorithms for 3D soft tissue landmarking on 3D photographs, but these algorithms did not include deep learning models. Baksi et al. described an automated method, involving morphing of a template mesh, for the landmarking of 22 soft-tissue landmarks from 3D photographs that achieved a precision of 3.2 ± 1.6 mm^11^. An automated principal component analysis-based method, described by Guo et al., achieved an average root mean square error of 1.7 mm for the landmarking 17 soft-tissue landmarks from 3D photographs^12^.

Studies that did apply deep learning for landmark detection on 3D meshes mostly focused on landmarking on intra-oral scans. Wu et al. utilized a two-stage deep learning framework for the prediction of 44 dental landmarks and achieved a mean absolute error of 0.623 ± 0.718 mm^21^. DentalPointNet, consisting of two sub-networks; a region proposal network and a refinement network, as described by Lang et al.^22^, achieved an average localization error of 0.24 mm for the detection of 68 landmarks. Even though the precision achieved in these studies is better than was achieved in this study, a direct comparison is infeasible to make due to the difference in landmarks, datasets, object size, and imaging modalities used. Therefore, future research should investigate the efficacy of these methods for cephalometric soft-tissue landmarking on 3D photographs. However, as the precision achieved by this method (1.69 ± 1.15 mm) closely aligns with the inter-observer variability (1.31 ± 0.91 mm) observed for the ten landmarks utilized, it is improbable that employing an alternative machine learning-based method trained and evaluated on the same dataset would yield large improvements in precision. Especially beyond the inter-observer variability, as the evaluation of the developed method is confined to the precision level dictated by the inter-observer variability of the manual annotation method.

The effect of landmark choice on established precision is underlined by the MeshMonk results found in this study. In the original publication by White et al., an average error of 1.26 mm for 19 soft-tissue landmarks was seen^10^. The same methodology was used to establish the precision for the ten landmarks used in this study, and an overall precision of 1.97 ± 1.34 mm was found. This finding highlights the difficulty in comparing landmarking precision from literature. Compared to the semi-automatic method, the fully-automated workflow yielded significantly improved precision for six landmarks, emphasizing the feasibility of fully-automatically annotating soft-tissue landmarks from 3D photos using deep learning.

The proposed workflow uses two successive networks and additional algorithms for alignment and facial segmentation. Advantages of this approach include that the DiffusionNet assures robustness against sampling densities and the HKS settings inherently account for rotational, positional, and scale invariance that may arise between different 3D photography systems. Input resolution can be considered a limiting factor for the maximum landmark detection performance. This can partially explain the differences as seen in the examples mentioned earlier (e.g. intra-oral scans have a much higher mesh resolution as compared to e.g. 3D photos or CT-scans). Furthermore, as DiffusionNet natively gives a per vertex prediction, the precision of the landmarking method is dependent on the vertex density of the 3D meshes. Therefore, the original high resolution meshes were used for the refined landmark prediction step. Since the achieved precision exceeded half the inter-vertex distance, it is expected that the impact of the inter-vertex distance on the precision was negligible. Moreover, employing the weighted mean vertex location for transforming the predicted vertices into landmark coordinates ensures that the predicted landmarks are not constrained solely to the vertex coordinates of the mesh.

A limitation of the current study is that the workflow was only applied to 3D photographs captured using one 3D photography system. Despite the robust nature of DiffusionNet/HKS, the performance of the workflow might be affected when applied to 3D photographs captured with different hardware. Furthermore, the DiffusionNet models were only trained on spatial features, whereas in the manual annotation process texture information was used. Even though this has the advantage of making the DiffusionNet models insensitive to variations in skin tone or color, landmarks such as the exocanthions, endocanthions, and cheilions could presumably be located more precisely using manual annotation. This would not apply to the landmarks lacking color transitions, such as the nasion and nose tip. Based on these presumptions, the DiffusionNet-based approach might achieve a better precision if texture data of the 3D photographs would be available to the networks.

Another limitation of the proposed workflow arises from the utilization of HKS settings in the initial DiffusionNet, leading to occasional issues with random left–right flipping in the predictions of symmetrical landmarks (e.g., exocanthions). To overcome this challenge, a solution was devised that involved detecting symmetrical landmarks within a single channel. Subsequently, both landmarks were distinguished from each other using a clustering algorithm, followed by a left–right classification based on the midsagittal plane. Although a success rate of 98.6% was achieved using this solution, the workflow failed when the initial DiffusionNet was unable to predict one of the landmarks in the midsagittal plane (nasion, nose tip, or cheilion midpoint). Since this was mainly due to suboptimal quality of the 3D photo, it might be prevented by optimizing image acquisition. For optimal performance of the workflow, it is important to minimize gaps and restrict the depicted area in 3D photos to the face.

Within this workflow, ten clinically relevant yet rather easily identifiable and reproducible landmarks, were incorporated to provide a solid gold standard. However, the constraint of utilizing only ten landmarks may impede the adaptability of the current method to a broader anatomical region or specific research objectives. The effectiveness of the developed workflow for the detection of more challenging landmarks should be investigated in future research. However, adapting the current workflow to accommodate additional landmarks or different anatomical regions may necessitate modifications.

Due to its high precision and consistency, the developed automated landmarking method has the potential to be applied in various fields. Possible applications include objective follow-up and analysis of soft-tissue facial deformities, growth evaluation, facial asymmetry assessment, and integration in virtual planning software for 3D backward planning^23,24^. Considering that the proposed DiffusionNet-based approach only uses spatial features, it could be applied on 3D meshes of facial soft-tissue that are derived from imaging modalities lacking texture, such as CT, CBCT, or MRI. Nevertheless, further research is necessary to ascertain the applicability of this workflow to these imaging modalities. The fully-automated nature of the workflow also enables cephalometric analysis on large-scale datasets, presenting significant value for research purposes. The position-independency of the workflow might make it suitable for automated landmarking in 4D stereophotogrammetry and give rise to real-time cephalometric movement analysis for diagnostic purposes^25,26^.

## Conclusion

In conclusion, a deep learning-based approach for automated landmark extraction from 3D facial photographs was developed and its precision was evaluated. The results showed high precision and consistency in landmark annotation, comparable to manual and semi-automatic annotation methods. Automated landmarking methods offer potential for analyzing large datasets, with applications in orthodontics, genetics, and craniofacial surgery and in emerging new imaging techniques like 4D stereophotogrammetry.

### Supplementary Information

Supplementary Information.

## Data Availability

Coded scripts are available within the following GitHub repository: https://github.com/rumc3dlab/3dlandmarkdetection/.
