# Supplementary material for: Fully automated landmarking and facial segmentation on 3D photographs
Source: Sci Rep. 2024 Mar 18;14:6463. doi: 10.1038/s41598-024-56956-9 (PMC10948387; doi:10.1038/s41598-024-56956-9)
Supplement: Supplementary file 1 — Supplementary Information. [file 41598_2024_56956_MOESM1_ESM.docx]

**Appendix**

**Appendix Table 1.** The parameters set used for the configuration of the MeshMonk algorithm.

| **Parameters** | **Values** |
| --- | --- |
| **Rigid Registration** | |
| *Number of iterations* | 30 |
| *Correspondence neighbor number* | 3 |
| *Correspondence flag threshold* | 0.90 |
| *Correspondence symmetric* | Yes |
| *Correspondence equalize* | No |
| *Use scaling* | Yes |
| *Inlier kappa* | 4.00 |
| *Inlier use orientation* | Yes |
| *Floating boundary* | Yes |
| *Target boundary* | Yes |
| *Target badly shaped triangles* | Yes |
| *Triangle size Z factor* | 6.00 |
| *Target up sample* | No |
| ***Non-rigid registration*** | |
| *Number of iterations* | 80 |
| *Correspondence neighbor number* | 3 |
| *Correspondence flag threshold* | 0.90 |
| *Correspondence symmetric* | Yes |
| *Correspondence equalize* | No |
| *Inlier kappa* | 12.00 |
| *Inlier use orientation* | Yes |
| *Floating boundary* | Yes |
| *Target boundary* | Yes |
| *Target badly shaped triangles* | Yes |
| *Triangle size Z factor* | 6.00 |
| *Target upsample* | Yes |
| *Inlier use weights* | No |
| *Transform sigma* | 3.00 |
| *Viscous iteration start* | 200 |
| *Viscous iteration end* | 1 |
| *Elastic iteration start* | 200 |
| *Elastic iteration end* | 1 |
| *Transform neighbors* | 80 |
